# Physiology of ECG: A ‘Mannequin’ based approach for first year medical students

**DOI:** 10.30476/jamp.2020.85262.1175

**Published:** 2021-04

**Authors:** PUJA DULLOO, CHAITRI SHAH

**Affiliations:** 1 Department of Physiology, Pramukhswami Medical College, Bhaikaka University, Karamsad, Anand, Gujarat, India; 2 Department of Anesthesia, Ahmadabad, Gujarat, India

**Dear Editor**

In-depth understanding of physiology course can be acquired if students are provided opportunity to identify patho-physiology via clinical practicum ( 1 ). Simulators and mannequins’ have a positive impact towards providing better clinical understanding and have a feel of authentic ‘real’ clinical environment and experience ( 2 ), although it has less been implement in physiology program. Introduction of computer simulator programs for physiology laboratory has been appreciated and valued by various researchers ( 3 , 4 ). The aim of the study is to evaluate the perception and learning outcome among first year medical students for physiological basis of ECG, using mannequin based teaching approach.

Observational, cross sectional study commenced for 150 first year medical students of 2017-18 batch. The theoretical aspect of the ECG was taught during 3hrs lecture within three days, followed by 3hrs of practical session in the Labdhi (Skilled Lab) center and physiology lab of the Smt.B.K.Shah Medical institute & Research center, Sumandeep Vidyapeeth University.

A pre-test was conducted after theory lectures and post test after completion of the practical. Students were divided into 6 groups (25 students in each). ECGs presentations for normal and various cardiac and metabolic disorders were conducted on “Heartsim 200” mannequins. Clinical case series as practical practice sheets were discussed after the lab session, separately. 

Students perception for corrective implementation of mannequin based teaching was collected, by using a feedback survey questionnaire, based on five points Likert scale with two open-ended questions. Three Focus group discussions (FGD) were conducted (8 students in each group) after the session.

Present study shows statistical significance (Mean±SD;15.48±19.05; p<0.0001) and middle level of correlation (0.50; p<0.625), between pre and post test result of participants (N=97) implying enhanced learning outcome with this teaching approach. These results are aligned with that of the study conducted by other researcher ( 1 , 5 ).

Friedman ranking analysis for the feedback items showed maximum ranking for the question specifying that this technique was for deeper understanding of the concept (11.03), participants enjoyed applied basis for learning of physiology (11.02). Lowest rank for questions “The concepts learned during these sessions may not be useful in near future” (3.53) and “Every student of the class was actively engaged and involved for this program” (6.06). Gender variability was observed for some of the questions.

The study concludes that this teaching method encourages students to be in a “near real live setting environment” and have better clinical conceptual understanding of the basic science topic taught. Simulations based sessions are organizational skills which will prepare students for real patient setting without compromising the patient care and safety. 
